# Impact of hospital sepsis case volume on mortality in sepsis patients: a meta-analysis

**DOI:** 10.3389/fmed.2026.1777875

**Published:** 2026-02-16

**Authors:** Jiaan Chen, Fan Zhang, Da Sun, Guangjun Jin

**Affiliations:** 1 Department of Clinical Medicine, The Second Clinical Medical College, Zhejiang Chinese Medical University, Hangzhou, China; 2 Department of Emergency, The Second Affiliated Hospital of Zhejiang Chinese Medical University, Hangzhou, China

**Keywords:** hospital case volume, ICU mortality, in-hospital mortality, meta-analysis, sepsis

## Abstract

**Objective:**

Evidence regarding the impact of annual hospital sepsis case volume on clinical outcomes in patients with sepsis remains controversial. This study aimed to conduct a meta-analysis to evaluate the potential association between annual sepsis case volume and mortality among patients with sepsis.

**Methods:**

A comprehensive electronic search was performed in PubMed, Web of Science, Embase, and Cochrane Library databases. Mean differences (MDs) or odds ratios (ORs) and 95% confidence intervals (CIs) were calculated using Review Manager 5.3.

**Results:**

A total of 4,408,416 patients from 18 studies were included in this meta-analysis, comprising 1,828,689 patients treated in high-volume hospitals and 2,579,727 patients treated in low-volume hospitals. Compared with low-volume hospitals, treatment in high-volume hospitals was associated with significantly lower in-hospital mortality [OR = 0.90 (95% CI: 0.87–0.93, *P* < 0.00001)], ICU mortality [OR = 0.93 (95% CI: 0.91–0.94, *P* < 0.00001)], and early mortality [OR = 0.81 (95% CI: 0.76–0.87, *P* < 0.00001)], as well as a significantly shorter ICU length of stay [MD = −0.11 days (95% CI: −0.22 to −0.01, *P* = 0.04)]. However, no significant difference was observed in hospital length of stay between high- and low-volume hospitals.

**Conclusions:**

Hospitals with a high annual sepsis case volume are associated with reduced mortality among patients with sepsis. Future studies are warranted to further define clinically meaningful thresholds for high-volume hospitals.

## 1 Introduction

Sepsis has become one of the leading causes of in-hospital mortality worldwide. In the United States alone, sepsis accounts for approximately 19 million hospitalizations and more than 5 million deaths annually (1). It is estimated that sepsis imposes an economic burden of nearly USD 24 billion on the U.S. healthcare system each year (2). Consequently, effective strategies to reduce the clinical and economic burden of sepsis are urgently needed. Early identification, timely treatment, and optimization of care processes are widely recognized as key determinants of sepsis outcomes (2).

High-volume centers generally possess greater clinical experience and more abundant medical resources, which may facilitate timely evaluation and implementation of effective treatments. Therefore, treatment in high-volume hospitals may confer potential survival benefits for patients with sepsis (3–5). However, the association between hospital volume and sepsis outcomes remains controversial. Recently, a study by Ohki et al. (6) involving 934 patients with sepsis reported improved survival outcomes in high-volume centers compared with low-volume centers. In addition, Kahn et al. (7) estimated that routinely transferring patients from low-volume hospitals to high-volume hospitals across eight U.S. states could potentially save approximately 4,720 lives per year. In contrast, a study by Naar et al. (8) found no significant association between hospital volume and in-hospital mortality among patients with sepsis.

Given these conflicting findings, we systematically collected the currently available evidence and conducted a meta-analysis to investigate the association between hospital sepsis case volume and mortality outcomes in patients with sepsis.

## 2 Methods

### 2.1 Search strategy

This review was conducted in accordance with the Preferred Reporting Items for Systematic Reviews and Meta-Analyses (PRISMA) guidelines. The study protocol was registered in PROSPERO (CRD420261289937). PubMed, Web of Science, Embase, and the Cochrane Library were systematically searched from database inception to October 20, 2025. The complete search strategy is presented in Table 1. In addition, the reference lists of eligible studies were manually screened to identify potentially relevant articles. No language restrictions were applied.

**Table 1:** Electronic search strategy.

**Database**	**Search term (published up to October 20, 2025)**	**Number**
PubMed	(centrali^*^[Title/Abstract] OR hospital volume^*^[Title/Abstract] OR center volume^*^[Title/Abstract] OR center volume^*^[Title/Abstract] OR low volume^*^[Title/Abstract] OR lower volume^*^[Title/Abstract] OR lowest volume^*^[Title/Abstract] OR high volume^*^[Title/Abstract] OR higher volume^*^[Title/Abstract] OR highest volume^*^[Title/Abstract] OR medium volume^*^[Title/Abstract] OR mid volume^*^[Title/Abstract] OR middle volume^*^[Title/Abstract] OR minimum volume^*^[Title/Abstract] OR volume outcome^*^[Title/Abstract] OR surgical volume^*^[Title/Abstract] OR surgeon volume^*^[Title/Abstract] OR surgeon's volume^*^[Title/Abstract] OR volume cost^*^[Title/Abstract] OR case volume^*^[Title/Abstract] OR caseload volume^*^[Title/Abstract] OR patient volume^*^[Title/Abstract] OR procedure volume^*^[Title/Abstract] OR procedural volume^*^[Title/Abstract] OR volume standard^*^[Title/Abstract]) AND (sepsis[Title/Abstract] OR Pyemia [Title/Abstract] OR Pyohemia[Title/Abstract] OR septicemia[Title/Abstract] OR septic[Title/Abstract])	981
Embase	(centrali^*^ OR hospital volume^*^ OR center volume^*^ OR center volume^*^ OR low volume^*^ OR lower volume^*^ OR lowest volume^*^ OR high volume^*^ OR higher volume^*^ OR highest volume^*^ OR medium volume^*^ OR mid volume^*^ OR middle volume^*^ OR minimum volume^*^ OR volume outcome^*^ OR surgical volume^*^ OR surgeon volume^*^ OR surgeon's volume^*^ OR volume cost^*^ OR case volume^*^ OR caseload volume^*^ OR patient volume^*^ OR procedure volume^*^ OR procedural volume^*^ OR volume standard^*^).ab,kw,ti. AND (sepsis OR Pyemia OR Pyohemia OR septicemia OR septic).ab,kw,ti.	1,972
Cochrane library trials	((centrali^*^ OR hospital volume^*^ OR center volume^*^ OR center volume^*^ OR low volume^*^ OR lower volume^*^ OR lowest volume^*^ OR high volume^*^ OR higher volume^*^ OR highest volume^*^ OR medium volume^*^ OR mid volume^*^ OR middle volume^*^ OR minimum volume^*^ OR volume outcome^*^ OR surgical volume^*^ OR surgeon volume^*^ OR surgeon's volume^*^ OR volume cost^*^ OR case volume^*^ OR caseload volume^*^ OR patient volume^*^ OR procedure volume^*^ OR procedural volume^*^ OR volume standard^*^):ti,ab,kw) AND ((sepsis OR Pyemia OR Pyohemia OR septicemia OR septic):ti,ab,kw)	1,690
Web of science	(TS=(centrali^*^ OR hospital volume^*^ OR center volume^*^ OR center volume^*^ OR low volume^*^ OR lower volume^*^ OR lowest volume^*^ OR high volume^*^ OR higher volume^*^ OR highest volume^*^ OR medium volume^*^ OR mid volume^*^ OR middle volume^*^ OR minimum volume^*^ OR volume outcome^*^ OR surgical volume^*^ OR surgeon volume^*^ OR surgeon's volume^*^ OR volume cost^*^ OR case volume^*^ OR caseload volume^*^ OR patient volume^*^ OR procedure volume^*^ OR procedural volume^*^ OR volume standard^*^)) AND (TS=(sepsis OR Pyemia OR Pyohemia OR septicemia OR septic))	484

### 2.2 Study selection

Studies were eligible for inclusion if they met the following criteria: (1) Population: patients diagnosed with sepsis, severe sepsis, or septic shock; (2) Intervention: treatment in hospitals with a high annual sepsis case volume (high-volume hospitals); (3) Comparison: treatment in hospitals with a low annual sepsis case volume (low-volume hospitals). Hospital sepsis case volume was defined as the annual number of sepsis cases treated in the hospital during the year of patient admission; (4) Outcomes: the primary outcome was in-hospital mortality. Secondary outcomes included ICU mortality, early mortality, ICU length of stay, and hospital length of stay. Early mortality was defined as death within 2 days of admission; (5) Study type: randomized controlled trials, cohort studies or case-control studies.

Exclusion criteria were as follows: case reports, conference abstracts, animal studies, studies without relevant outcome indicators, and letters.

### 2.3 Data extraction

Data were independently extracted from all eligible studies by two authors (Jiaan Chen and Fan Zhang) using a standardized data extraction form. Disagreements were resolved through discussion with a third independent author (Guangjun Jin). Extracted data included author name, year of publication, sepsis definition, study design, country, study population (sample size and volume category), and outcome data (in-hospital mortality, ICU mortality, early mortality, ICU length of stay, and hospital length of stay). When required data were unavailable, corresponding authors were contacted for additional information.

### 2.4 Quality assessment

The quality assessment was conducted independently by two authors (Jiaan Chen and Fan Zhang) using the Newcastle-Ottawa Scale (NOS), which assigns a score on a nine-point scale. A score of ≥7 indicates high quality, and scores of 5–6 indicate moderate quality. Any discrepancies were resolved by a third author (Guangjun Jin).

### 2.5 Statistical analysis

Meta-analyses were performed using Review Manager (RevMan) version 5.3 (The Nordic Cochrane Center, The Cochrane Collaboration, Copenhagen, Denmark) and Stata version 14. Odds ratios (ORs) with corresponding 95% confidence intervals (CIs) were calculated for categorical outcomes (in-hospital mortality, ICU mortality, and early mortality), and mean differences (MDs) were calculated for continuous outcomes (ICU length of stay and hospital length of stay). Statistical heterogeneity was assessed using the Cochran *Q* test and the *I*^2^ statistic. A random-effects model was applied when *I*^2^ > 50%; otherwise, a fixed-effects model was used (9). Subgroup analyses were performed based on patient age and sepsis definition era (pre-2016 vs. post-2016). Sensitivity analyses were conducted using a one-study removal approach. Publication bias was assessed using funnel plots and Egger's test when more than 10 studies were available. A *P* value < 0.05 was considered statistically significant.

## 3 Results

### 3.1 Literature retrieval

**Figure 1:**
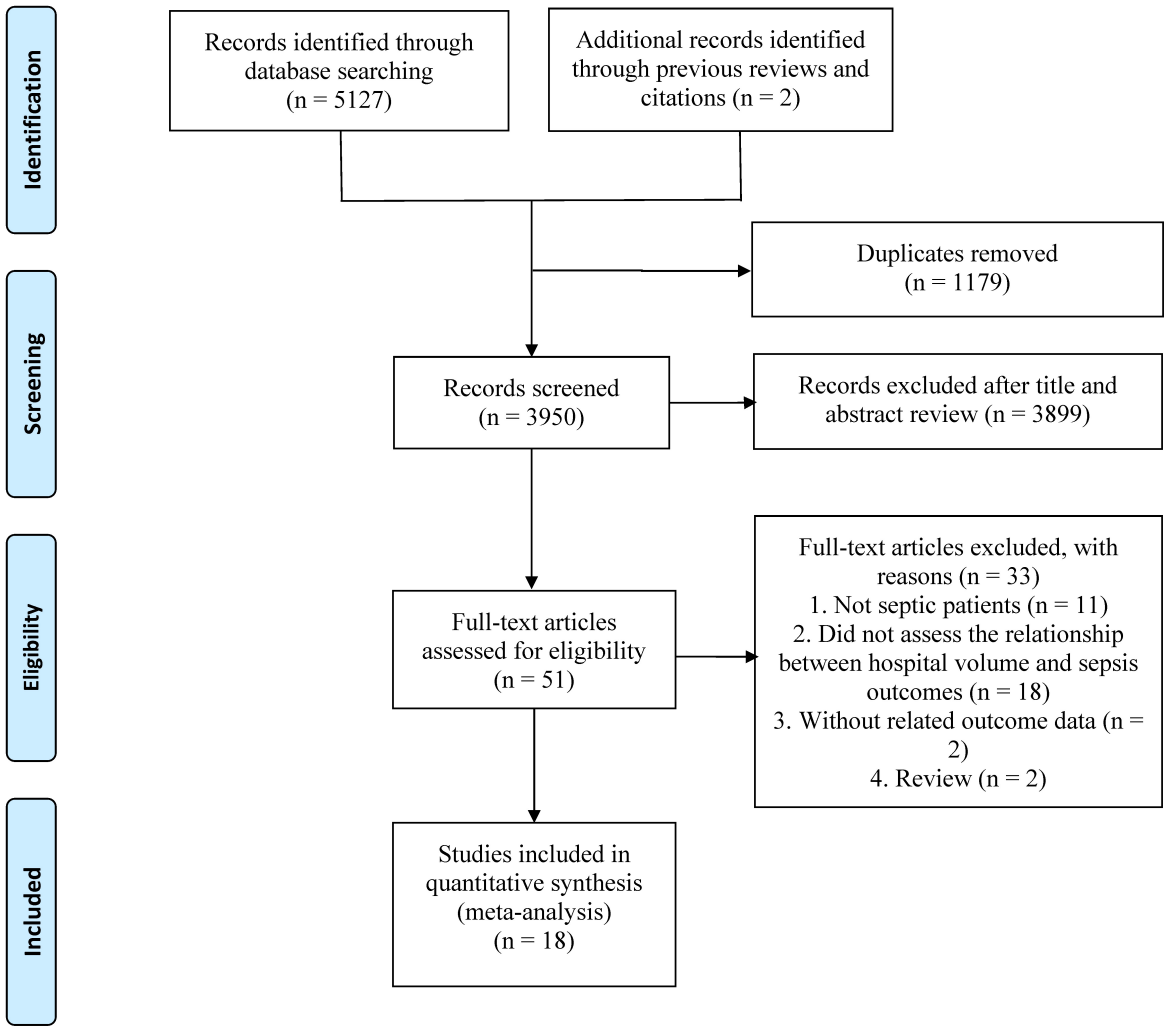
The PRISMA flowchart.

The initial database search identified 5,129 records (Figure 1), of which 1,179 were duplicates. After reviewing titles and abstracts, 3,899 papers were excluded, and the full texts of the remaining 51 studies were evaluated. Finally, 18 eligible studies (4–6, 8, 10–23) were enrolled in this study.

### 3.2 Study characteristics and quality assessment

The included studies were published between 2005 and 2025 and involved a total of 4,408,416 patients (high-volume hospitals: 1,828,689 patients; low-volume hospitals: 2,579,727 patients). Sample sizes ranged from 934 to 1,213,219 patients. All 18 studies were retrospective in design. The study populations were primarily drawn from the United States, France, China, Japan, and the United Kingdom. All studies were rated as moderate to high quality, with NOS scores ≥5. Detailed study characteristics are summarized in Table 2.

**Table 2:** Characteristics of eligible studies.

**First author, year**	**Design**	**Country or region**	**Period of study**	**Diagnostic criteria**	**Sample size**	**Patients in each group**	**Patients**	**Volume cut-off (case/year)**	**Outcome**	**NOS**
Markovitz 2005	RCS	USA	2001–2002	Severe sepsis	6,693	LVH: 1,504 HVH: 5,189	Pediatric sepsis	LVH: ≤ 250 HVH: > 250	In-hospital mortality	6/9
Powell 2010	RCS	USA	2007	Sepsis	87,166	LVH: 65,035 HVH: 22,131	Adult sepsis	LVH: ≤ 371 HVH: > 371	In-hospital mortality, early mortality	6/9
Banta 2012	RCS	USA	2005–2010	Severe sepsis	1,213,219	LVH: 1,010,458 HVH: 202,761	Adult sepsis	LVH: < 500 HVH: ≥ 500	In-hospital mortality	6/9
Shahin 2012	RCS	UK	2008–2009	Severe sepsis	30,720	LVH: 10,460 HVH: 20,260	Adult sepsis	LVH: ≤ 537 HVH: > 537	In-hospital mortality, ICU mortality, ICU length of stay, hospital length of stay	8/9
Zuber 2012	RCS	France	1997–2008	Septic shock	3,437	LVH: 299 HVH: 3,138	Adult sepsis	LVH: < 13 HVH: ≥ 13	ICU mortality, ICU length of stay, hospital length of stay	7/9
Walkey 2013	RCS	USA	2011	Severe sepsis	1,838	LVH: 199 HVH: 1,639	Adult sepsis	LVH: NA HVH: NA	In-hospital mortality, hospital length of stay	7/9
Gaieski 2014	RCS	USA	2004–2011	Severe sepsis	914,200	LVH: 319,596 HVH: 594,604	Adult sepsis	LVH: < 500 HVH: ≥ 500	In-hospital mortality	7/9
Kocher 2014	RCS	USA	2005–2009	Sepsis	528,767	LVH: 317,683 HVH: 211,084	Adult sepsis	LVH: NA HVH: NA	In-hospital mortality, early mortality	6/9
Shahul 2014	RCS	USA	2002–2011	Severe sepsis	646,988	LVH: 94,695 HVH: 552,293	Adult sepsis	LVH: < 60 HVH: ≥ 60	In-hospital mortality	7/9
Goodwin 2015	RCS	USA	2010	Severe sepsis	9,815	LVH: 497 HVH: 9,318	Adult sepsis	LVH: < 75 HVH: ≥ 75	In-hospital mortality, hospital length of stay	6/9
Fawzy 2017	RCS	USA	2010–2012	Sepsis	287,914	LVH: 129,148 HVH: 158,766	Adult sepsis	LVH: < 353 HVH: ≥ 353	In-hospital mortality, hospital length of stay	7/9
Maharaj 2021	RCS	UK	2010–2016	Sepsis	273,001	LVH: 138,221 HVH: 134,780	Adult sepsis	LVH: NA HVH: NA	In-hospital mortality, ICU mortality, hospital length of stay, ICU length of stay	7/9
Naar 2021	RCS	USA	2014–2015	Sepsis	10,716	LVH: 1,350 HVH: 9,366	Adult sepsis	LVH: NA HVH: NA	In-hospital mortality, ICU mortality	7/9
Chen 2022	RCS	China	2020	Septic shock case	1,920	LVH: 943 HVH: 959	Adult sepsis	LVH: < 74 HVH: ≥ 74	In-hospital mortality, ICU mortality	7/9
Fujinaga 2024	RCS	Japan	2015–2021	Sepsis	72,214	LVH: 55,065 HVH: 17,149	Adult sepsis	LVH: < 541 HVH: ≥ 541	In-hospital mortality	8/9
Oami 2024	RCS	Japan	2010–2017	Sepsis	317,365	LVH: 158,099 HVH: 159,266	Adult sepsis	LVH: < 198 HVH: ≥ 198	In-hospital mortality, hospital length of stay, ICU length of stay	8/9
Scott 2024	RCS	USA	2015–2021	Sepsis	1,527	LVH: 760 HVH: 767	Pediatric sepsis	LVH: < 100 HVH: ≥ 100	In-hospital mortality	7/9
Ohki 2025	RCS	Japan	2014–2018	Sepsis	934	LVH: 700 HVH: 234	Pediatric sepsis	LVH: < 26 HVH: ≥ 26	In-hospital mortality	8/9

HVH, high-volume hospitals; LOS, length of stay; LVH, low-volume hospitals; ICU, intensive care unit; NOS, Newcastle-Ottawa Scale; RCS, retrospective cohort study; UK, United Kingdom; USA, United States of America.

### 3.3 Meta-analysis

#### 3.3.1 In-hospital mortality

**Figure 2:**
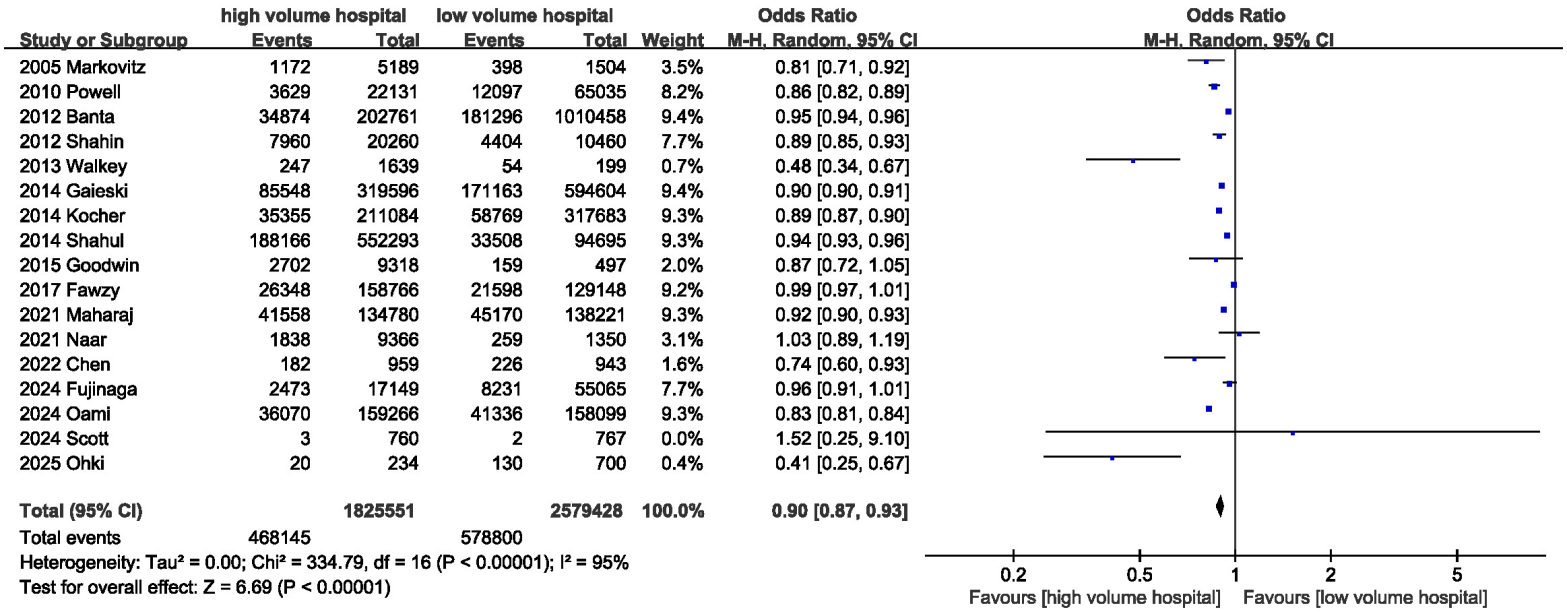
Meta-analysis of in-hospital mortality between high volume hospital and low volume hospital.

Seventeen studies reported data on in-hospital mortality. The pooled results of the 17 studies showed that in-hospital mortality was significantly lower in high-volume hospitals compared with low-volume hospitals [OR = 0.90 (95% CI: 0.87–0.93, *P* < 0.00001)] (Figure 2; Table 3); however, substantial heterogeneity was observed among the included studies (*I*^2^ = 95%; *P* < 0.00001).

**Table 3:** Summary of results of meta-analysis.

**Outcomes of interest**	**Studies, *n***	**Events for high volume hospital**	**Events for low-volume hospitals**	**MD/OR**	**95%CI**	***P*-value**	***I*^2^ (%)**
In-hospital mortality	17	468,145/1,825,551	578,800/2,579,428	0.90	0.87, 0.93	< 0.00001	95
ICU mortality	5	38,933/168,503	36,093/151,273	0.93	0.91, 0.94	< 0.0001	24
Early mortality	2	14,791/233,215	28,806/382,718	0.81	0.76, 0.87	< 0.0001	77
ICU length of stay	4	–	–	−0.11	−0.22, −0.01	0.04	89
Hospital stay	7	–	–	0.35	−0.71, 1.41	0.51	99

CI, confidence interval; ICU, intensive care unit; MD, mean differences; OR, Odds ratio.

#### 3.3.2 ICU mortality

**Figure 3:**
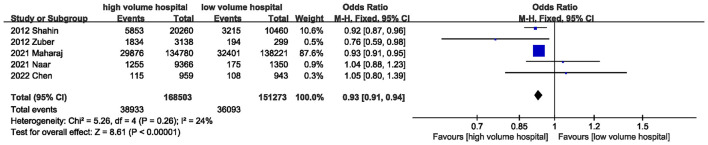
Meta-analysis of ICU mortality between high volume hospital and low volume hospital.

Five studies assessed ICU mortality. The pooled results indicated that treatment in high-volume hospitals was associated with a lower risk of ICU mortality [OR = 0.93 (95% CI: 0.91–0.94, *P* < 0.00001)] (Figure 3; Table 3).

#### 3.3.3 Early mortality

**Figure 4:**
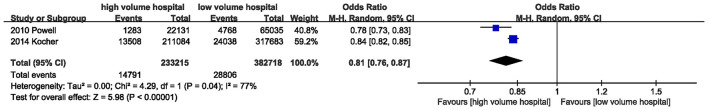
Meta-analysis of early mortality between high volume hospital and low volume hospital.

Early mortality was evaluated in two studies, and the pooled results showed that early mortality was lower in high-volume hospitals than in low-volume hospitals [OR = 0.81 (95% CI: 0.76–0.87, *P* < 0.00001)] (Figure 4; Table 3).

#### 3.3.4 Hospital stay

**Figure 5:**
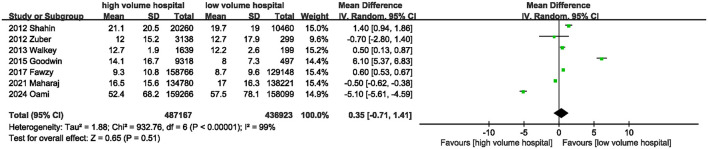
Meta-analysis of hospital stay between high volume hospital and low volume hospital.

Seven studies provided information on hospital stay. The combined results showed that the high-volume hospital group had a similar hospital length of stay to the low-volume hospital group [MD = 0.35 days (95% CI: −0.71 to 1.41, *P* = 0.51)] (Figure 5; Table 3).

#### 3.3.5 ICU length of stay

**Figure 6:**
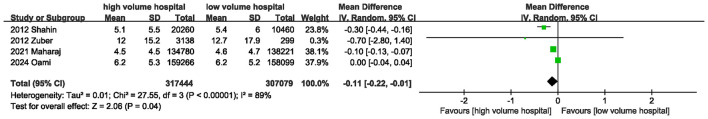
Meta-analysis of ICU length of stay between high volume hospital and low volume hospital.

The ICU length of stay was reported in 4 trials. The pooled results indicated that treatment in high-volume hospitals was associated with a significantly shorter ICU length of stay compared with low-volume hospitals [MD = −0.11 days (95% CI: −0.22 to −0.01, *P* = 0.04)] (Figure 6; Table 3).

### 3.4 Subgroup analysis

**Figure 7:**
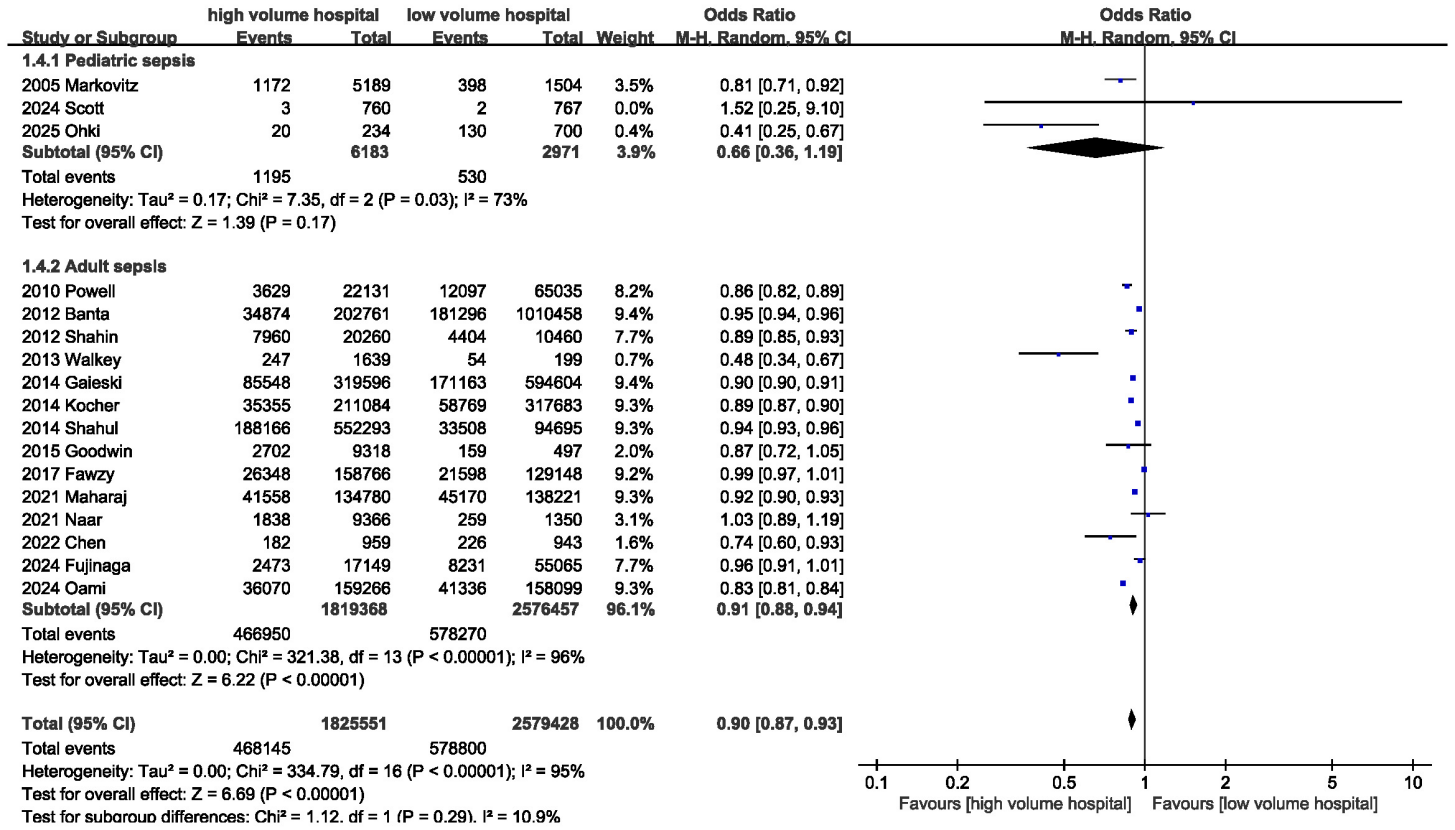
Subgroup analysis of in-hospital mortality between high volume hospital and low volume hospital.

Subgroup analyses were conducted based on age and sepsis definition era (Table 4). In adult patients, treatment in high-volume hospitals was associated with significantly lower in-hospital mortality [OR = 0.91 (95% CI: 0.88–0.94, *P* < 0.00001)] (Figure 7). In pediatric patients, although mortality was numerically lower in high-volume hospitals, the difference did not reach statistical significance [OR = 0.66 (95% CI: 0.36–1.19, *P* = 0.17)]. Moreover, in both the pre-2016 and post-2016 sepsis definition subgroups, treatment in high-volume hospitals was consistently associated with reduced in-hospital mortality and ICU mortality. However, no substantial reduction in heterogeneity for in-hospital mortality was observed across these subgroups.

**Table 4:** Summary of results from subgroup analyses.

**Indicators**	**Subgrouped by**	**The number of studies**	**Effect size**	**95%CI**	***I*^2^ (%)**	***P* for between subgroup heterogeneity**
In-hospital mortality	Age	–	–	–	–	0.29
	Adult sepsis	14	0.91	0.88, 0.94	96	–
	Pediatric sepsis	3	0.66	0.36, 1.19	73	–
	Sepsis definition era	–	–	–	–	0.92
	Pre-2016	9	0.90	0.87, 0.93	92	–
	Post-2016	8	0.90	0.84, 0.98	97	–
ICU mortality	Sepsis definition era	–	–	–	–	0.35
	Pre-2016	2	0.91	0.86, 0.96	51	–
	Post-2016	3	0.93	0.92, 0.95	15	–
ICU length of stay	Sepsis definition era	–	–	–	–	0.003
	Pre-2016	2	−0.30	−0.44, −0.16	0	–
	Post-2016	2	−0.05	−0.15, 0.05	93	–
Hospital stay	Sepsis definition era	–	–	–	–	0.01
	Pre-2016	4	1.91	−0.47, 4.30	98	–
	Post-2016	3	−1.62	−3.06, −0.18	100	–

ICU, intensive care unit.

### 3.5 Publication bias and sensitivity analysis

**Figure 8:**
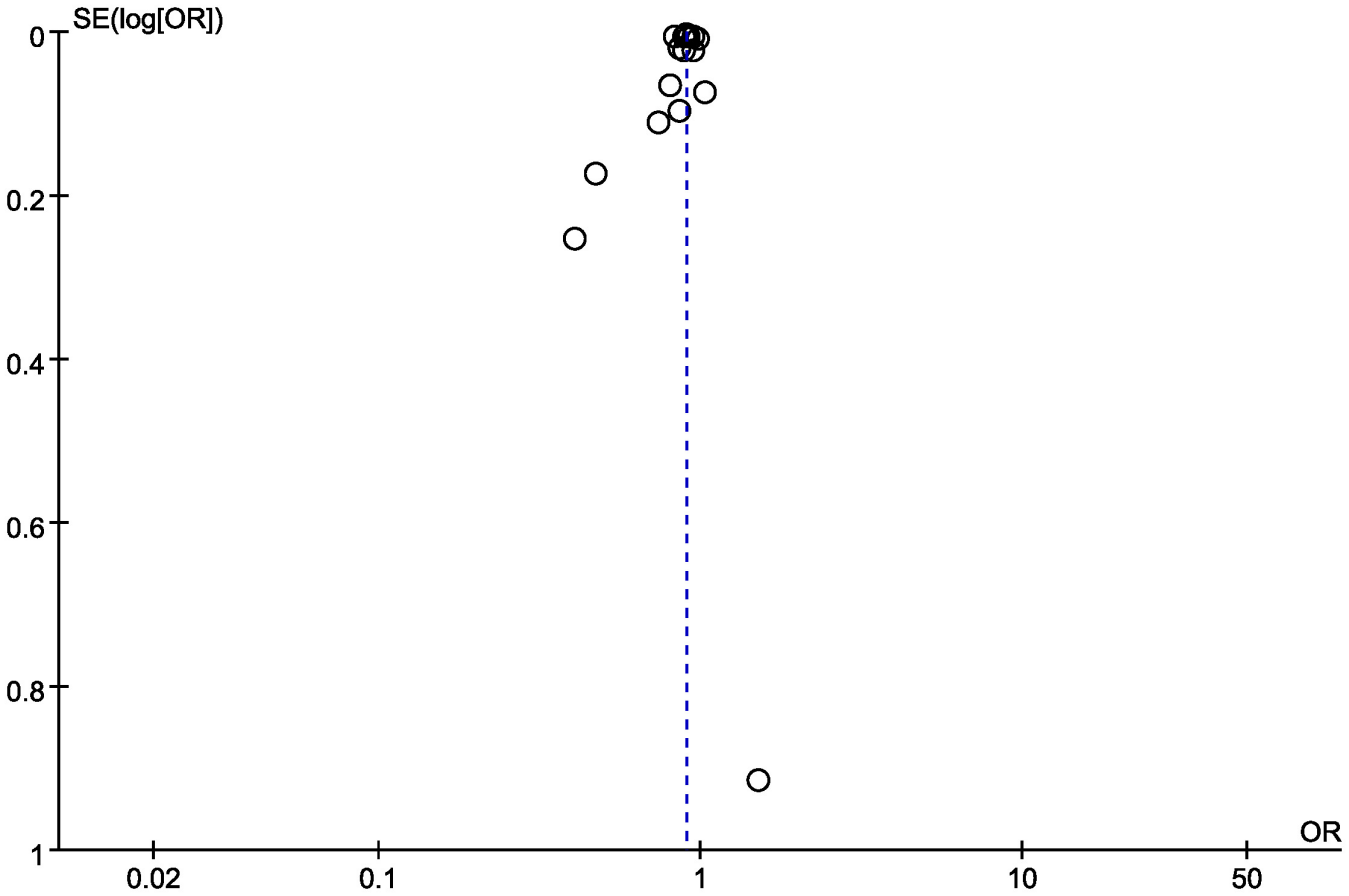
Funnel plot of in-hospital mortality between high volume hospital and low volume hospital.

Egger's test (*P* = 0.519) and funnel plot inspection indicated no significant publication bias for in-hospital mortality (Figure 8). Sensitivity analyses showed that no single study substantially influenced the pooled estimates for in-hospital mortality, ICU mortality, early mortality, or ICU length of stay. However, removal of the study by Oami et al. resulted in a notable change in the pooled estimate for hospital length of stay (MD, 1.32 days; 95% CI, 0.45, 2.20).

## 4 Discussion

Over the past two decades, substantial evidence has evaluated the association between hospital volume and clinical outcomes in patients with sepsis; however, the findings remain inconsistent. Gu et al. (24) conducted a meta-analysis of nine studies published before 2015 and reported an inverse association between annual case volume and mortality among patients with sepsis [OR = 0.76 (95% CI: 0.65–0.89, *P* = 0.001)]. Nevertheless, in recent years, advances in medical technology and evolving sepsis management strategies have substantially altered clinical practice. Therefore, data from more recently published studies may better reflect contemporary real-world care.

In comparison, our meta-analysis incorporated more up-to-date evidence, including 18 studies encompassing a total of 4,408,416 patients. In addition, we evaluated a broader range of clinically relevant outcomes, including in-hospital mortality, ICU mortality, and early mortality. Our findings demonstrate that higher hospital sepsis volume is associated with lower in-hospital mortality, ICU mortality, and early mortality. Moreover, whereas previous meta-analyses focused exclusively on adult patients with sepsis, our study included a wider age spectrum, encompassing both adult and pediatric populations. Age-stratified subgroup analyses showed that higher hospital volume was significantly associated with reduced in-hospital mortality in adult patients. In pediatric patients, although in-hospital mortality was lower in high-volume centers compared with low-volume centers, the difference did not reach statistical significance. These findings have important clinical implications, as they provide evidence supporting an association between higher hospital volume and improved survival outcomes in patients with sepsis, which may help inform healthcare policy and clinical decision-making.

The relationship between hospital volume and clinical outcomes has been well-documented in several medical fields. In complex surgical procedures, such as pancreatic resection, colorectal surgery, pancreaticoduodenectomy, aortic dissection repair, kidney transplantation, and cardiac surgery, high-volume centers consistently demonstrate superior outcomes compared with low-volume centers (25–30). A meta-analysis by Fischer et al. (26) confirmed the positive impact of hospital volume on outcomes following pancreatic surgery. Similarly, Guo et al. (25) reported reduced postoperative mortality after colorectal cancer surgery in high-volume hospitals. Weng et al. (29) found that lower surgeon case volume in kidney transplantation was associated with an increased risk of severe sepsis and graft failure, including mortality. Mortality remains a critical outcome measure in sepsis management, with previous data indicating that in-hospital mortality rates for sepsis remain as high as 26.7% (5). Our results suggest that in-hospital mortality among patients with sepsis is significantly lower in high-volume centers, consistent with several prior studies (2, 31, 32). Ofoma et al. (2) demonstrated that patients with severe sepsis treated in high-volume hospitals had the lowest mortality. Wang et al. (31) assessed healthcare capacity using three ICU volume-related indicators (the proportion of septic shock patients occupying ICU beds, the patient-to-intensivist ratio, and the patient-to-nurse ratio), and found that treatment in hospitals with greater care capacity was associated with lower in-hospital mortality among patients with septic shock. Immunosuppressive conditions are well-established risk factors for mortality in patients with severe infections. Greenberg et al. (32) reported that among patients with sepsis complicated by immunosuppressive diseases, mortality risk was significantly higher in low-volume hospitals than in high-volume hospitals. With regard to the length of hospital stay, no significant difference was observed between high-volume and low-volume hospitals. Although a statistically significant reduction in ICU length of stay was noted in high-volume centers compared with low-volume centers, the magnitude of this reduction was modest, and its clinical relevance may therefore be limited.

Although the hospital volume–outcome relationship was first reported in 1979, the mechanisms underlying the association between higher hospital volume and improved survival in sepsis remain incompletely understood (24). Several potential explanations have been proposed. First, higher patient volumes may enable hospitals to accumulate greater clinical experience, with frequent patient encounters facilitating continuous process optimization and quality improvement, thereby delivering higher-quality care (24). Second, differences in care processes may exist between high- and low-volume centers. Early implementation of standardized sepsis treatment bundles has been associated with lower in-hospital mortality, and adherence to these protocols tends to be higher in high-volume centers, suggesting that more consistent application of evidence-based care may contribute to improved outcomes (6). Third, differences in institutional preparedness may play a role. A U.S.-based study demonstrated that higher emergency department preparedness scores were associated with improved survival, and high-volume centers are more likely to achieve higher preparedness ratings (6, 33). Finally, high-volume hospitals often possess greater healthcare resources, including higher staffing levels, more advanced medical equipment, and improved access to multidisciplinary care teams, all of which may contribute to better sepsis outcomes (4, 34). Ofoma et al. (34) evaluated hospitals based on six resource utilization characteristics (bed capacity, annual sepsis volume, major diagnostic procedures, renal replacement therapy, mechanical ventilation, and major therapeutic interventions), and classified hospitals into low-, medium-, and high-capacity tiers. Lower-capacity hospitals may be better suited to managing less complex sepsis cases.

From a clinical practice perspective, these findings suggest that centralization of sepsis care may help improve patient outcomes (24). However, comprehensive centralization is challenging to implement, particularly in rural or sparsely populated regions. Moreover, the risk–benefit balance of such strategies requires further investigation. In addition to centralization, a tiered care approach may represent a feasible alternative, whereby low-volume centers manage patients with milder disease, while more severe or complex cases are identified early and transferred to high-volume centers. The expanding application of internet-based healthcare systems may further transform current care models by fostering collaborative networks between high- and low-volume centers. Remote multidisciplinary consultations and decision-support guidance may improve outcomes in patients with complex sepsis. In the future, artificial intelligence–assisted tools may also provide valuable decision support for clinicians practicing in low-volume or resource-limited settings (35).

Our study has several strengths. On the one hand, we conducted a comprehensive literature search across multiple databases, thereby minimizing potential selection bias. On the other hand, we focused exclusively on patients with sepsis, ensuring a relatively homogeneous and clinically relevant study population.

This study has the following limitations. First, substantial heterogeneity was observed among the included studies, which may be attributable to differences in study design, regional variations in ICU staffing expertise and experience, healthcare system structures, and definitions of hospital volume. To account for these factors, random-effects models were applied when heterogeneity was high. We also performed age-based subgroup analyses; although higher hospital volume was associated with lower mortality in pediatric patients, the difference did not reach statistical significance. Given the limited number of studies evaluating pediatric sepsis, this finding warrants further investigation. Moreover, variations in the definition of sepsis across studies may represent a potential source of heterogeneity. Given that the Sepsis-3 definitions were updated in 2016, we performed subgroup analyses based on the era of sepsis definition. The results demonstrated that, in both the pre-2016 and post-2016 sepsis definition subgroups, treatment in high-volume hospitals was associated with reduced in-hospital mortality and ICU mortality. No significant heterogeneity was observed between the two subgroups with respect to in-hospital mortality. Second, most included studies were retrospective in nature and therefore subject to inherent limitations of observational designs. Additionally, several studies did not exclude transferred patients; low-volume hospitals are more likely to refer severe or complex cases to high-volume centers, which may attenuate the observed benefits of high-volume care. Nonetheless, our findings still indicate a survival advantage in high-volume centers. Finally, data regarding the impact of hospital volume on long-term outcomes were lacking. Given the observed association between hospital volume and in-hospital mortality, further studies are needed to explore the relationship between hospital volume and long-term outcomes in patients with sepsis.

In conclusion, based on the most recent and currently available evidence, this meta-analysis demonstrates that higher hospital volume is associated with a reduced risk of in-hospital mortality among adult patients with sepsis. However, the relationship between hospital volume and outcomes in pediatric sepsis requires further investigation. Future studies should aim to elucidate the mechanisms underlying the volume–outcome relationship and to identify clinically meaningful volume thresholds associated with improved survival.
